# Conditional knockout of TGF-βRII /Smad2 signals protects against acute renal injury by alleviating cell necroptosis, apoptosis and inflammation

**DOI:** 10.7150/thno.35686

**Published:** 2019-10-21

**Authors:** Qin Yang, Gui-ling Ren, Biao Wei, Juan Jin, Xiao Ru Huang, Wei Shao, Jun Li, Xiao-ming Meng, Hui Yao Lan

**Affiliations:** 1 The Key Laboratory of Major Autoimmune Diseases, Anhui Institute of Innovative Drugs, School of Pharmacy, Anhui Medical University; The key laboratory of Anti-inflammatory and Immune Medicines, Ministry of Education, Hefei 230032, China.; 2 Department of Pharmacy, The 901 Hospital of Chinese People's Liberation Army Joint Service Support Unit, Hefei, China; 3 Department of Medicine & Therapeutics, Li Ka Shing Institute of Health Sciences, and Shenzhen Research Institute, The Chinese University of Hong Kong, Hong Kong, China; 4 School of Basic Medical Sciences, Anhui Medical University. Anhui, China; 5 The Key Laboratory of Major Autoimmune Diseases, Anhui Institute of Innovative Drugs, School of Pharmacy, Anhui Medical University; The key laboratory of Anti-inflammatory and Immune Medicines, Ministry of Education, Hefei 230032, China.

**Keywords:** Acute kidney injury, Cisplatin, TGF-β receptor, Smad2, Necroptosis, Inflammation, Apoptosis

## Abstract

**Rationale**: TGF-β/Smad signaling is the central mediator for renal fibrosis, however, its functional role in acute kidney injury (AKI) is not fully understood. We previously showed Smad2 protects against renal fibrosis by limiting Smad3 signaling, but details on its role in acute phase are unclear. Recent evidence showed that TGF-β/Smad3 may be involved in the pathogenesis of AKI, so we hypothesized that Smad2 may play certain roles in AKI due to its potential effect on programmed cell death.

**Methods**: We established a cisplatin-induced AKI mouse model with TGF-β type II receptor or Smad2 specifically deleted from renal tubular epithelial cells (TECs). We also created stable *in vitro* models with either Smad2 knockdown or overexpression in human HK2 cells. Importantly, we evaluated whether Smad2 could serve as a therapeutic target in both cisplatin- and ischemic/reperfusion (I/R)-induced AKI mouse models by silencing Smad2 *in vivo*.

**Results**: Results show that disruption of TGF-β type II receptor suppressed Smad2/3 activation and attenuated renal injury in cisplatin nephropathy. Furthermore, we found that conditional knockout of downstream Smad2 in TECs protected against loss of renal function, and alleviated p53-mediated cell apoptosis, RIPK-mediated necroptosis and p65 NF-κB-driven renal inflammation in cisplatin nephropathy. This was further confirmed in cisplatin-treated Smad2 knockdown and overexpression HK2 cells. Additionally, lentivirus-mediated Smad2 knockdown protected against renal injury and inflammation while restoring renal function in established nephrotoxic and ischemic AKI models.

**Conclusions**: These findings show that unlike its protective role in renal fibrosis, Smad2 promoted AKI by inducing programmed cell death and inflammation. This may offer a novel therapeutic target for acute kidney injury.

## Introduction

Transforming growth factor-β (TGF-β) plays critical roles in cell growth, differentiation, extracellular matrix deposition and immune response 1, 2. Of note, its function in fibrogenesis has been extensively studied 3, 4. When released from LAP (latency-associated peptide) and LTBP (latent TGF-β-binding protein), active TGF-β1 binds with its type II receptor that activates type I receptor and downstream effectors, Smad2 and Smad3, to regulate genes associated with renal fibrosis 3.

Acute kidney injury (AKI) is a clinical syndrome characterized by a sudden decline in renal function 5-7. The common pathological features of AKI are programmed cell death of resident renal cells and excessive inflammation 6, 8-11. The role of TGF-β in AKI has drawn significantly more attention recently but is still unclear. Previous studies indicated a protective role for TGF-β in AKI; knockout of TGF-β1 aggravated ischemic kidney injury in mice 12. And, TGF-β protected against hydrogen peroxide-induced necrosis in tubular epithelial cells (TECs) 13. However, a recent study with conditional knockout of TGF-β1 type II receptor (TGF-βRII) in mice showed disrupted TGF-β1 signaling attenuated renal damage by reducing tubular apoptosis in mercuric chloride-induced injury 14. This was further confirmed by a separate study that showed activation of TGF-β signaling by overexpression of TGF-β1 type I receptor (TGF-βRI) induced renal cell apoptosis, necrosis, oxidative stress and interstitial infiltration of inflammatory cells 14, 15. In addition, inhibition of TGF-β1 increased proliferation of kidney tubular epithelial cells (TECs) while promoting repair after renal ischemia/reperfusion (I/R) injury 16. In this setting, more detailed studies have focused on the functional role of downstream Smads. Interestingly, one group found that knockout of Smad3 attenuated renal damage by limiting renal inflammation in a murine model of ischemic AKI 17. Our group also found that Smad7 knockout mice suffered more severe renal impairment; Smad7 rescued TGF-β/Smad3/p21/p27-induced G1 cell cycle arrest of TECs 18. Although we previously reported that Smad2 protected against Smad3-mediated renal fibrosis, no evidence regarding the role of Smad2 in AKI has been reported 19. In this study, our result showed that conditional knockout of TGF-βRII from TECs attenuated Smad2/3 activation and protected against renal injury caused by cisplatin injection, we further found Smad2 aggravated cisplatin-induced AKI *in vivo* using Smad2 conditional knockout mice, which was further confirmed* in vitro* using cisplatin-challenged TECs with Smad2 overexpression (OE) or knockdown (KD). More importantly, the therapeutic potential of Smad2-targeted strategy has been tested in both nephrotoxic and ischemic AKI models.

## Methods

### Regents

Cisplatin were obtained from Sigma-Aldrich (Sigma, CA, USA). Annexin V-FITC/PI Apoptosis Detection Kit was purchased from Beyotime (Shanghai, China). Periodic acid Schiff (PAS), Creatinine Assay Kit and BUN Assay Kit were obtained from Nanjing Jiancheng Bioengineering Institute (Nanjing, China). Fetal bovine serum (FBS), DMEM, and other cell culture reagents were purchased from Invitrogen. Antibodies specific to KIM-1, GAPDH, P-p53, p53, P-p65, p65 were purchased from Santa Cruz Biotechnology (Santa Cruz, CA, USA). RIPK1 and RIPK3 were obtained from BOSTER Biological Technology (Wuhan, China). F4/80+, anti-cleaved caspase-3, TNF-ɑ, P-Smad3, Smad3 and Smad2 were obtained from Cell Signaling Technology (CST, Danvers, MA). IRDye 800-conjugated secondary antibody was obtained from Li-cor biosciences (NE, USA). Lipofectamine 3000 was purchased from SciencBio Technology (Invitrogen, BeiJing, China).

### Generation of TGF-β type II receptor and Smad2 conditional KO Mice

All the animal experiments were approved by the Ethics Committees of the Chinese University of Hong Kong and all procedures were performed under the permission of the Guideline of Animal Care and Use Committee of the Chinese University of Hong Kong. TGF-β type II receptor kidney-specific conditional knockout mice were generated by mating the TGF-βRII FF mouse (C57B/L6) with the KspCre (C57B/L6) mouse as we previously reported 20. Smad2 kidney-specific conditional knockout mice were generated by mating the Smad2FF mouse (C57B/L6) with the KspCre (C57B/L6) mouse. The Smad2 flox mouse and KspCre transgenic mouse were generated and verified as described previously 19. Conditional deletion of the Smad2 gene from the kidney TECs was testified as we previously reported 19.

### Establishment of cisplatin-induced AKI Model

Animal model of cisplatin-induced AKI was established in groups of TGF-βRII FF and TGF-βRII KspCre mice, Smad2FF and S2FF/KspCre mice (male, 8 weeks of age, 22 to 25 g) by intraperitoneal injection of cisplatin (20 mg/kg) respectively. Mice intraperitoneal injected with saline were used as control. All mice were generated from the genetically identical littermates, and all animals were sacrificed under anesthesia 3 days after injection. Kidney tissue samples were harvested for Periodic acid Schiff (PAS) Staining, immunohistochemistry, Western blot and Real-Time PCR as reported previously 21, and blood samples were harvested for BUN and Creatinine detection in accordance with manufacturer's instructions.

### Establishment of I/R-induced AKI Model

Mice were obtained from Laboratory Animal Center of Anhui province. All animal procedures were approved by the Institutional Animal Experimentation Ethics Committee of Anhui Medical University. All animal used were C57BL/6N mice (aged 6-8 weeks). Throughout the surgical procedure, the body temperature was maintained between 35 and 37.5 ℃. Mice were anesthetized, sterilized, and shaved. We performed a midline abdominal incision and a bilateral renal pedicle clipping. Both renal pedicles were clamped for 40 minutes with a microvascular clamp. After removing the clamp, reperfusion was confirmed visually. The abdomen was closed in two layers using standard 6-0 sutures. Sham-operated mice received identical surgical procedures, except that clamps were not applied. Twenty-four hours after IRI, blood and kidney samples from the mice were harvested for further study.

### Lentivirus-Mediated Smad2 Knockdown in Mice

Mouse Smad2 shRNA were obtained from GenePharm (Shanghai, China). Lentivirus-mediated Smad2 knockdown in mice was performed as we previously reported 21, then mice were intraperitoneally injected with either 20 mg/ kg cisplatin or the equal volume of saline or ischemia-reperfusion surgery and sham-operation for further analysis.

### Transfection of Smad2 small hairpin RNA and Smad2 overexpression plasmid constructs

Smad2 expression was assessed by Western blot and Real-time PCR analysis after transfection of HK2 cells with human-derived Smad2 shRNA or overexpression (OE) plasmid obtained from Genepharma Co., Ltd. Briefly, cells were seeded in 6-well plates and transfected with Smad2 shRNA/Smad2 OE plasmid and control constructs using Lipo3000 transfection reagent (Invitrogen, Carlsbad, CA, USA). Cells were incubated with opti-MEM at 37°C and 5% CO_2_ for 6 h. Cells were cultured in DMEM containing 5% FBS and selected by puromycin or G418 to establish Smad2 KD and OE stable cell lines.

### Cell Culture

Cells were cultured in HyClone™ DMEM-F12 medium-containing 5% FBS at 37℃ and 5% CO_2_. Cells were starved for 12 hours with 0.5% FBS, then treated with cisplatin (20 uM) for 24 hours. Cells were harvested and analyzed for indexes of tubular injury, inflammation, necroptosis, apoptosis, activation of NF-κB, p53, RIPK and Smad3 signaling by Real-Time PCR, Western blot analysis and responsive promoter assay. A minimum of 3 independent experiments were performed.

### Renal RNA Extraction and Real-Time PCR Examination

Total RNA was obtained from kidney tissues or cultured cells using the RNeasy Isolation Kit according to the manufacturer's instructions (Qiagen, Valencia, CA). Concentration of RNA was measured by a NanoDrop 2000 Spectrophotometer (Thermo scientific, USA). Total RNA was reverse transcribed into cDNA according to the manufacturer's instructions of Bio-Rad kit. Real-time PCR mixture contained 0.3 ul upstream and downstream primers for each gene, 4 μl Bio-Rad iQ SYBR Green supermix with Opticon2 (Bio-Rad, Hercules, CA), 2.4 ul enzyme-free water, and 2 ul cDNA solution. The sequences of primers, including mouse and human, were used as described previously 21-23. The sequences of other primers were as follows: human Smad2 (forward, 5'-ACTAACTTCCCAGCAGGAAT-3'; reverse, 5'-GTTGGTCACTTGTTTCTCCA-3'); human GAPDH (forward, 5'-CATGAGAAGTATGACAACAGCCT-3'; reverse, 5'-AGTCCTTCCACGATACCAAAGT-3'); mouse Smad2 (forward 5'-ATGTCGTCCATCTTGCCATTC-3'; reverse, 5'-AACCGTCCTGTTTTCTTTAGCTT-3'); mouse GAPDH (forward, 5'-TGCTGAGTATGTCGTGGAGTCTA-3'; reverse, 5'-AGTGGGAGTTGCTGTTGAAATC-3'). Real-time PCR Assay reaction conditions were: denaturation at 95 °C for 20 seconds, annealing at 58 °C for 20 seconds, elongation at 72 °C for 20 seconds, amplification for 40 cycles for each primer. GAPDH was used to normalize the ratio for the mRNA of other genes.

### Western Blot Analysis

Protein from kidney tissues and cultured cells was extracted with RIPA-Buffer (Beyotime, Jiangsu, China); BCA kit (Beyotime, Jiangsu, China) was used to measure concentration. Western blot was performed as described previously 19, 21. The membrane was incubated with 5% milk in PBS for two hours to block nonspecific binding, then incubated with appropriate antibody using rabbit anti-KIM-1, anti-P-p65/p65, anti-P-p53/p53, anti-cleaved casapase-3, anti-RIPK1, anti-RIPK3, anti-P-Smad3/Smad3 and mouse anti-GAPDH overnight at 4°C. Membrane was incubated with IRDye 800-conjugated secondary antibody for 2 hours at room temperature (Rockland immunochemicals). Images were captured with LiCor/Odyssey infrared image system (LI-COR Biosciences, Lincoln, NE) and then analyzed by Image J software (NIH, Bethesda, MD, USA).

### Periodic acid shiff staining and Immunohistochemical analysis

Periodic Acid Schiff (PAS) staining was performed with a PAS kit to assess the histological damage. The score of proximal renal impairment show extent of tubular necrosis and tubular dilatation as follows: 0=normal; 1=10%; 2=10%-25%; 3=26%-50%; 4=51%-75%; 5=75%-95%; 6=more than 96%. Immunohistochemistry was performed on paraffin sections to detect kidney injury by microwave antigen retrieval techniques. Sections were incubated with rabbit anti-KIM-1, anti-F4/80+, and anti-TNF-α antibody overnight at 4 °C. Sections were incubated in secondary antibody and chromagen liquid DAB (3, 30-diaminobenzidine tetrahydrochloride). Non-immune rabbit IgG was used as a negative control. After immunostaining, the slides were counterstained with hematoxylin. The results were quantitatively analyzed by Image Analysis System (AxioVision 4, Carl Zeiss, Jena, Germany) as described previously 21.

### Renal function detection

Blood samples from mice were used to measure creatinine and blood urea nitrogen (BUN) using Creatinine and BUN Assay Kits as previously described 21.

### Flow cytometric analyses

Flow cytometric analysis was performed to evaluate the percentage of apoptotic cells. The stable Smad2 knockdown HK2 cell lines were treated with or without 20uM cisplatin for 24 h. HK2 cells were digested with trypsin for 2 minutes and centrifuged at 1500 rpm for 5 minutes. According to the manufacturer's instructions, the density of cells used was 10^6^ cells/ml after addition of 400 ul Annexin V binding fluid. Cells were re-stained with 10ul PI for 5 minutes and lightly placed at 4 °C in dark before being immediately measured with a laser eight-color flow cytometer (FACSVerse, BD, USA) and quantified using FlowJo 7.6 software 21.

### Luciferase reporter assay

Luciferase reporter assay were performed according to the manufacturer's instructions as we previously descripted (Promega Corporation, WI, USA)^23^. p53, Smad3, p65 NF-κB luciferase reporters, contained p53, Smad3, p65 NF-κB binding sites in the promoter region of luciferase respectively, were designed and purchased from Genomeditech Co. Ltd (Genomeditech, Shanghai, China). In brief, Smad2 wild type and knockdown Smad2 HK2 cells were transiently transfected with p53, Smad3 or p65-responsive promoter Luc respectively. Cisplatin (20 uM) was added to the cells for 24 hours after transfection and starving for 12 hours. According to the manufacturer's instructions, the p53, Smad3, p65 NF-κB activities were analyzed by luciferase reporter gene assay. Their activities were normalized to promoter activity of normal group. 3-4 independent experiments were performed.

### Co-immunoprecipitation

HK2 cells were harvested by 1%NP-40 followed by centrifugation at 3000 rpm for 5 min. Protein was incubated with p53 antibody for 2 h at 4 °C. Then capture the immunocomplex by adding 100 μL of washed Protein A agarose bead slurry (EMD Millipore Corporation, 28820 Single Oak Drive, Temecula, CA 92590, USA). The tagged protein was incubated with the bead for 12 h at 37°C to get the protein-bead complex. Protein-bead complex was washed with three cycles of 1% NP-40. Samples were finally measured by Western blot with Smad2 antibody.

### TUNEL assay

Renal apoptosis was examined by TUNEL assay using the One step TUNEL Apoptosis Assay Kit from Beyotime Biotechnology (Beyotime, Jiangsu, China). Briefly, paraffin-embedded renal tissue sections were deparaffinized and added Proteinase K (20 μg/ml) 30 min at 37 ℃. The sections were then exposed to the TUNEL reaction mixture containing TM red-labeled dUTP. TUNEL-positive nuclei were identified by fluorescence microscopy.

### Statistical Analysis

The data acquired from this study are presented as the mean ± SEM from 3-4 independent *in vitro* experiments or 6-8 mice. Statistical analyses were performed using two-tailed unpaired *t* test or one-way ANOVA, followed by Newman-Keuls post hoc test (Prism 5.0; GraphPad Software, San Diego, CA).

## Results

### Generation and verification of conditional Smad2 KO mice, conditional TGF-βRII KO mice, stable Smad2 knockdown *in vitro and in vivo*, and Smad2 overexpression in tubular epithelial cell lines

**Figure 1:**
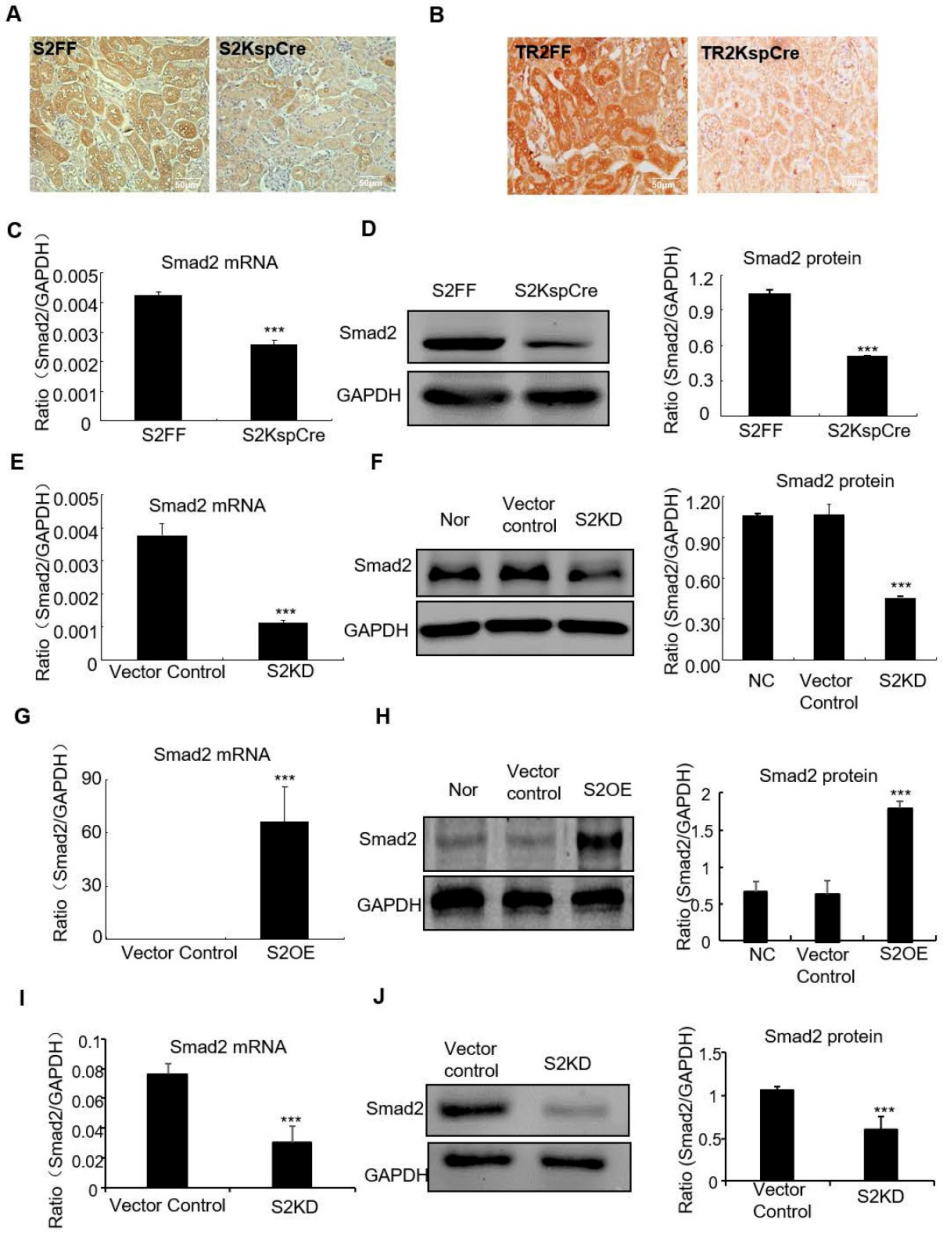
** Verification of conditional Smad2 and TGFβRII KO in vivo, stable Smad2 knockdown and Smad2 overexpression in HK2 cells.** A and B: Immunohistochemistry show Smad2 and TGFβRII deleted in kidney. C and D: Real-time PCR and Western blot show Smad2 deleted in kidney. E and F: Real-time PCR and Western blot show Smad2 knocked down from HK2 cells. G and H: Real-time PCR, Western blot and quantitative data show overexpression of Smad2 in HK2 cells. I and J: Real-time PCR and Western blot show Smad2 silence in mice. Data represent the mean ± SEM for groups of 6-8 mice *in vivo* and 3-4 independent experiments* in vitro*. ***P<0.001 compared to S2FF mice or Smad2 vector control. S2FF: Smad2 flox/flox mouse; S2 KspCre: conditional Smad2 knockout mice; S2KD: Smad2 knockdown; S2OE: Smad2 overexpression; TGF-βRII FF: TGF-βRII flox/flox mouse; TGF-βRII KspCre: conditional TGF-βRII knockout mice.

Smad2 conditional knockout mice (Smad2ff/KspCre) were generated by mating Smad2 flox/flox mice (Smad2FF) with kidney-specific promoter (Cadherin-16)-driven Cre mice (KspCre). Smad2 was silenced in kidney tubular epithelial cells. Immunohistochemistry show Smad2 and TGF-βRII were depleted from renal tubulars (Figures 1A and B). Real-time PCR results show Smad2 mRNA significantly decreased in kidneys of conditional Smad2 KO mice (Figure 1C). Western blot and quantitative data confirm protein levels reduced (Figure 1D). Results show Smad2 knockdown and overexpression in HK2 cell lines were successfully established (Figures 1E-H). Finally, Real-time PCR and Western blot results show Smad2 was successfully silenced in mice transfected with lentivirus-packaged Smad2 shRNA plasmid (Figures 1I and J).

### TGF-β promoted cisplatin-induced renal injury, inflammation and programmed cell death through TGF-β/Smads pathway

**Figure 2:**
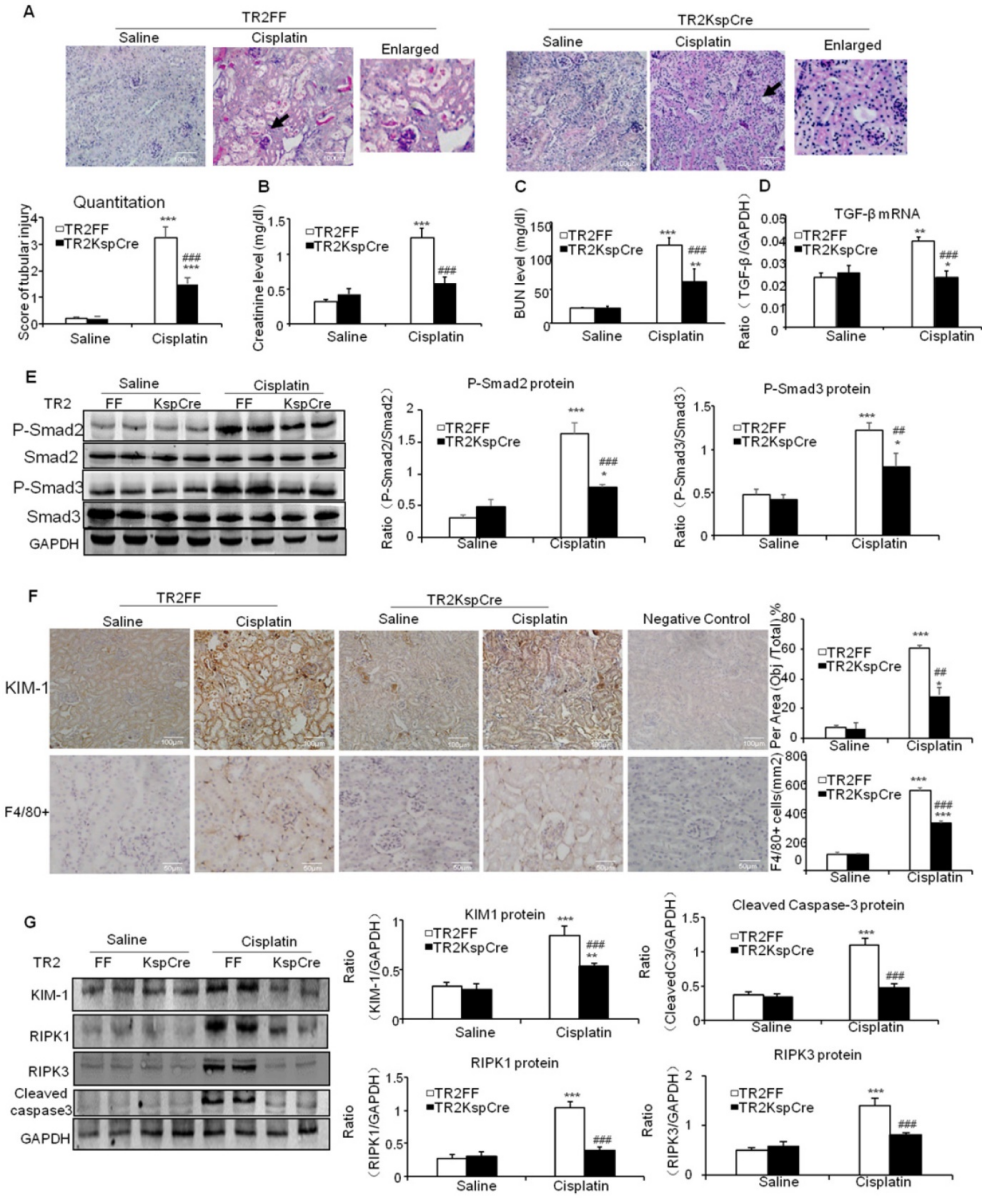
** Conditional knockout of TGF-βRII prevented cisplatin-induced renal injury and apoptotic signaling *in vivo*.** A: Periodic acid-Schiff (PAS) staining and quantitative analysis show conditional knockout of TGF-βRII reduced renal injury in cisplatin nephropathy. B: Creatinine assay. C: BUN assay. Serum creatinine and BUN show conditional knockout of TGF-βRII prevented decline of renal function in cisplatin nephropathy. D. Real-time PCR data show conditional knockout of TGF-βRII reduced TGF-β mRNA level in cisplatin-induced nephropathy. E. Western blot analysis of phospho-Smad2 and phospho-Smad3. F. Immunohistochemistry and quantitative data show conditional knockout of TGF-βRII reduced KIM-1 protein and F4/80+ macrophages infiltration in cisplatin nephropathy. G. Western blot analysis of KIM-1, RIPK1, RIPK3, cleaved caspase-3. Data represent mean ± SEM for 6-8 mice. **P<0.01, ***P<0.001 versus normal; ^##^P<0.01, ^###^P<0.001 versus TGF-βRII FF+ cisplatin group. TGF-βRII FF: TGF-βRII flox/flox mouse; TGF-βRII KspCre: conditional TGF-βRII knockout mice; Magnification: 100X.

PAS staining and quantitative data show inhibition of TGF-β signaling by conditional knockout of TGF-βRII from tubular epithelial cells attenuated kidney injury at day 3 after intraperitoneal injection of cisplatin (20 mg/kg) (Figure 2A). Results of creatinine and BUN assay consistently show conditional knockout of TGF-βRII protected against cisplatin-induced renal dysfunction (Figures 2B and C). Real-time PCR and Western blot results further show conditional knockout of TGF-βRII reduced TGF-β1 mRNA and suppressed Smad2/3 phosphorylation without altering total Smad2/3 protein in cisplatin nephropathy (Figure 2D and E). Additionally, disruption of TGF-β signaling suppressed the induction of KIM-1, F4/80+ macrophage infiltration and activation of key signaling molecules regulating programmed cell death (Figure 2F and G).

### Conditional knockout of Smad2 attenuated cisplatin-induced renal injury and programmed cell death *in vivo*

**Figure 3:**
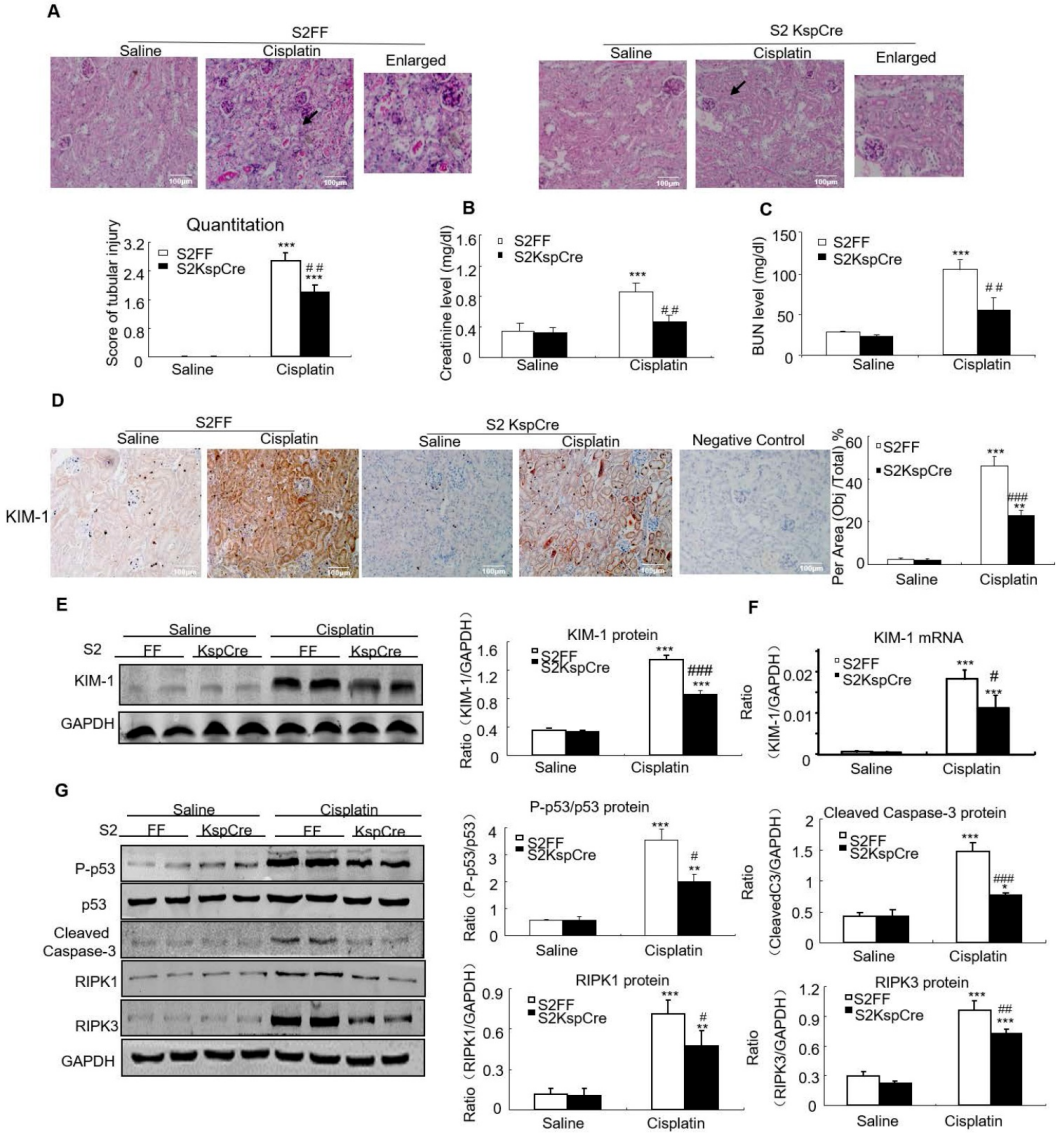
** Conditional knockout of Smad2 prevented cisplatin-induced renal injury, decline of renal function and attenuated signaling molecules regulating programmed cell death *in vivo*.** A: PAS staining and quantitative analysis show conditional knockout of Smad2 reduced renal injury in cisplatin-induced AKI mice. B: Creatinine assay. C: BUN assay. Serum creatinine and BUN show conditional deletion of Smad2 prevented decline of renal function in cisplatin nephropathy. D: Immunohistochemistry and quantitative data show conditional knockout of Smad2 reduced KIM-1 in cisplatin-induced nephropathy. E and F: Western blot and Real-time PCR analysis of KIM-1. G: Western blot of P-p53, p53, RIPK1, RIPK3 and cleaved caspase-3. Data represent mean ± SEM for 6-8 mice. **P<0.01, ***P<0.001 versus normal; ^#^P<0.05, ^##^P<0.01, ^###^P<0.001 versus Smad2FF+cisplatin group. S2FF: Smad2 flox/flox mouse; S2 KspCre: conditional Smad2 knockout mice; KIM-1: kidney injury molecule-1. Magnification: 100X.

PAS staining and quantitative data show disruption of Smad2 attenuated kidney damage in cisplatin nephropathy (Figure 3A). Results of creatinine and BUN assay also show conditional knockout of Smad2 prevented decline in renal function (Figures 3B and C). We also measured KIM-1 level to evaluate the function of Smad2 in AKI. Immunohistochemical results show KIM-1 significantly lower in Smad2 KspCre mice compared with S2FF mice (Figure 3D). And, KIM-1 mRNA and protein levels were significantly downregulated after conditional deletion of Smad2 in response to cisplatin (Figures 3E and F). Western blot results show cleaved caspase-3 and p53 phosphorylation significantly reduced after conditional knockout of Smad2 in cisplatin nephropathy. Additionally, disruption of Smad2 also reduced RIPK1 and RIPK3, the central regulators in necroptosis in cisplatin nephropathy (Figure 3G).

### Conditional knockout of Smad2 limited cisplatin-induced renal inflammation *in vivo*

**Figure 4:**
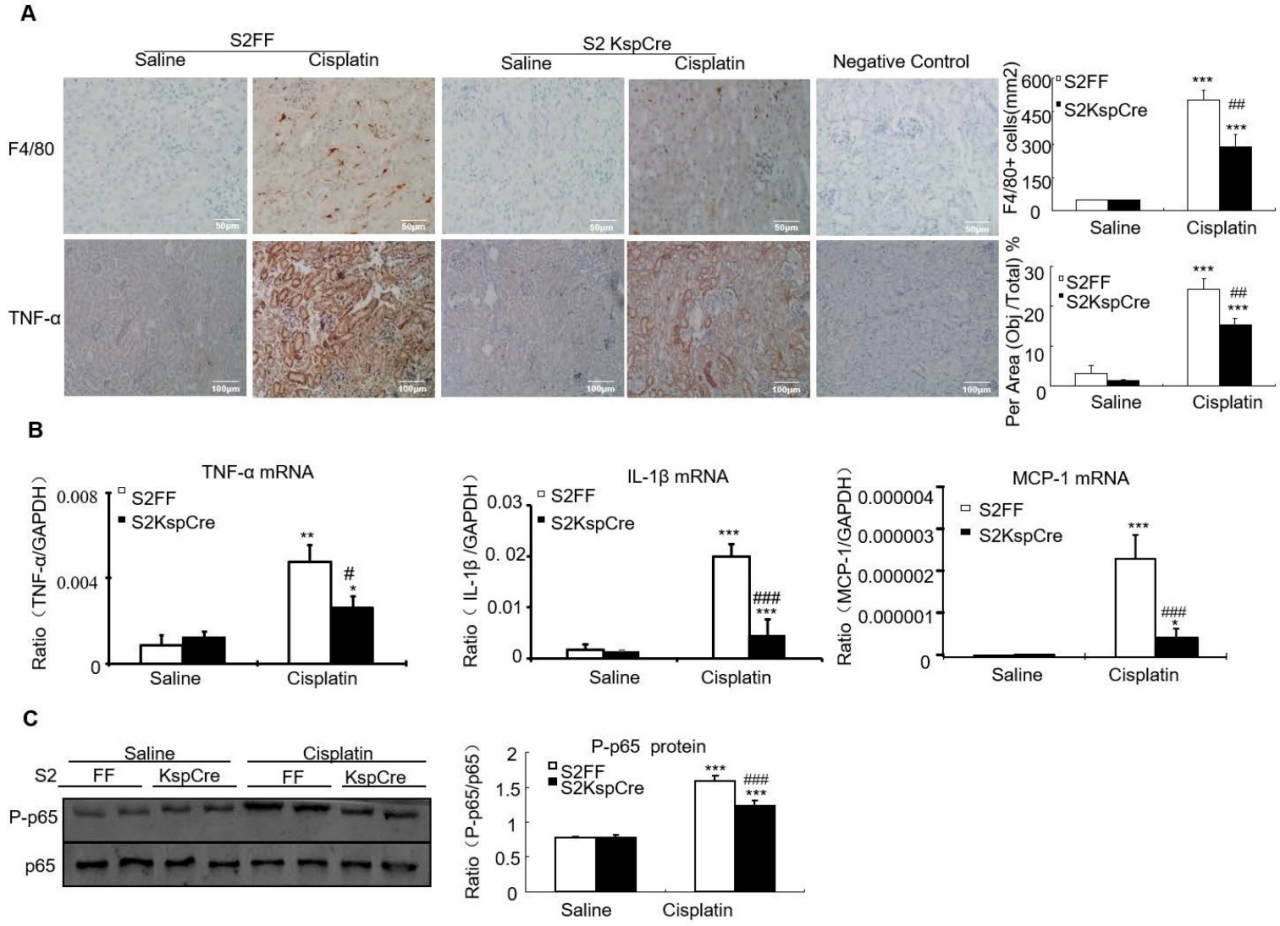
** Conditional knockout of Smad2 attenuated cisplatin-induced renal inflammation by suppressing p65 signaling *in vivo*.** A: Immunohistochemistry and quantitative data show conditional knockout of Smad2 reduced TNF-α protein and F4/80+ macrophages in cisplatin-induced nephropathy. B: Real-time PCR for inflammation indexes in mice. Conditional knockout of Smad2 reduced mRNA of TNF-α, IL-1β and MCP-1 compared with S2FF model group. C: Western blot analysis of p-P65 and P65. Data represent mean ± SEM for 6-8 mice. *P<0.05, **P<0.01, ***P<0.001 versus normal; ^#^P<0.05, ^##^P<0.01, ^###^P<0.001 versus Smad2FF+cisplatin group. S2FF: Smad2 flox/flox mouse; S2 KspCre: conditional Smad2 knockout mice; KIM-1: kidney injury molecule-1. Magnification: 100X.

Results of immunohistochemistry and quantitative data show that conditional deletion of Smad2 decreased F4/80+ macrophage infiltration by 40% compared with the Smad2FF control. Smad2 conditional knockout mice also had reduced expression of tumor necrosis factor-α (TNF-α). This was further confirmed by Real-time PCR show that mRNA levels of inflammatory indexes TNF-α, interleukin-1β (IL-1β) and chemokine monocyte chemotactic protein-1 (MCP-1) reduced in Smad2 conditional knockout mice (Figures 4A and B). Finally, conditional knockout of Smad2 prevented p65 NF-κB phosphorylation in kidneys of mice with AKI (Figure 4C).

### Knockdown of Smad2 alleviated cisplatin-induced renal injury, inflammation and programmed cell death *in vitro*

**Figure 5:**
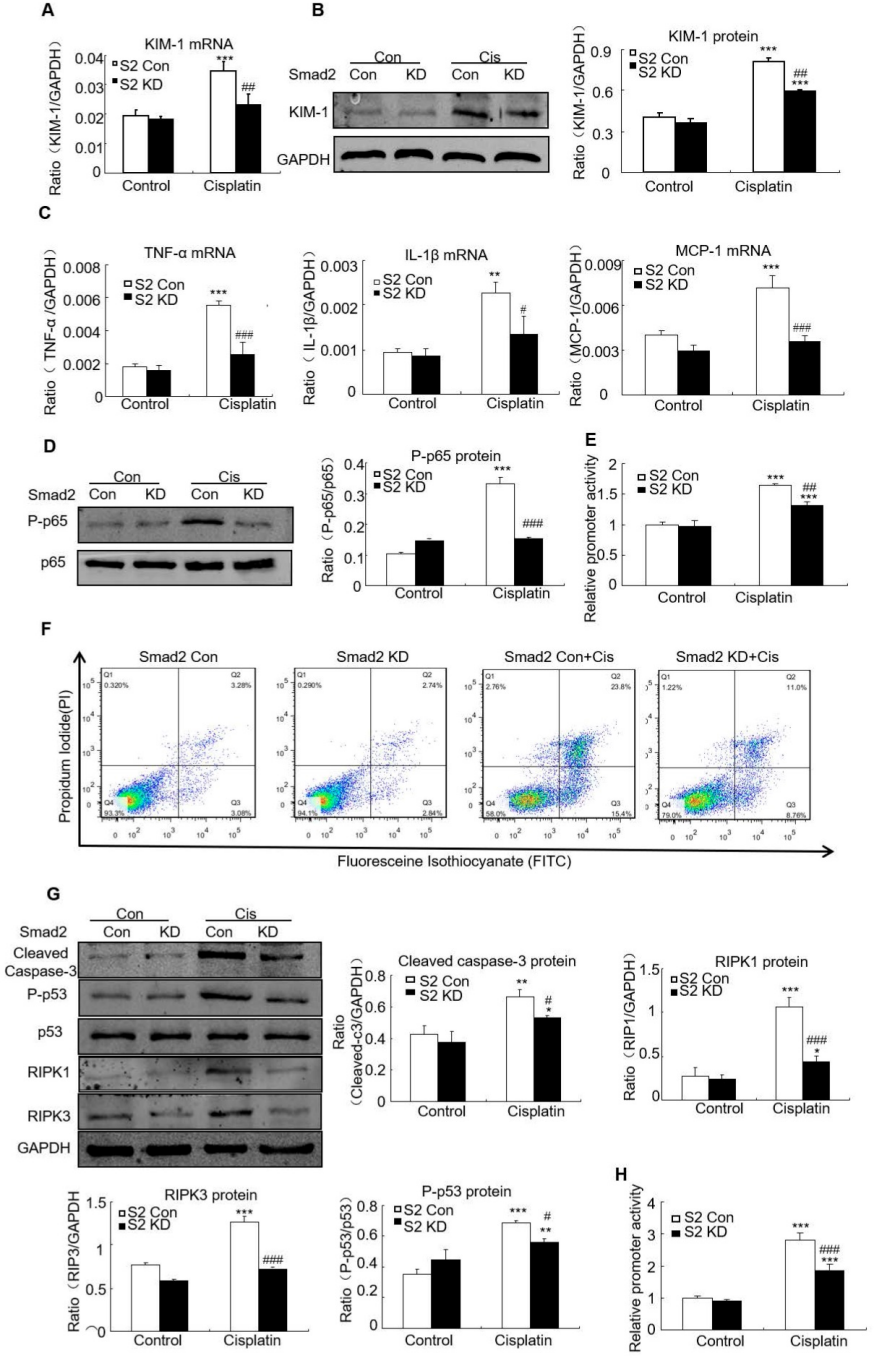
** Knockdown of Smad2 reduced cisplatin-induced injury, inflammation response and programmed cell death in HK2 cells.** A: Real-time PCR analysis of KIM-1 shows knockdown of Smad2 reduced KIM-1 mRNA in cisplatin-treated HK2 cells. B: Western blot and quantitative data show knockdown of Smad2 decreased KIM-1 protein. C: Real-time PCR analysis of inflammation indexes shows knockdown of Smad2 reduced cisplatin-induced inflammation response, including mRNA level of TNF-α, IL-1β and MCP-1. D: Western blot analysis of phospho-p65 in HK2 cells shows knockdown of Smad2 reduced cisplatin-induced p65 NF-κB phosphorylation. E: p65 NF-κB luciferase reporter assay. F: Flow cytometry of Smad2 knockdown HK2 cells shows decreased percentage of apoptotic and necrotic cells in cisplatin-treated HK2 cells. G: Western blot analysis of key molecules regulating programmed cell death. H: p53 luciferase reporter assay shows knockdown of Smad2 reduced cisplatin-induced p53 activity in HK2 cells. Data represent the mean ± SEM for 3-4 independent experiments. *P<0.05, **P<0.01, ***P<0.001 versus control; ^#^P<0.05, ^##^P<0.01, ^###^P<0.001 versus Smad2 vector control+cisplatin. KD: knockdown.

Real-time PCR and Western blot results show KIM-1 mRNA and protein levels significantly reduced in cisplatin-stimulated Smad2 KD cells (Figures 5A and B). Real-time PCR show mRNA levels of inflammatory indexes TNF-α, IL-1β and MCP-1 significantly reduced after Smad2 knockdown (Figure 5C). In addition, silence of Smad2 reduced phospho-p65 NF-κB, this was further confirmed by NF-κB luciferase reporter assay (Figures 5D and E). Moreover, loss of Smad2 reduced the programmed cell death of HK2 cells compared with cisplatin-treated group (Figure 5F). Knockdown of Smad2 reduced cleaved caspase-3, Phospho-p53, RIPK1 and RIPK3 level (Figure 5G). Additionally, luciferase reporter assay results show disruption of Smad2 largely reduced cisplatin-induced p53 activity compared with control HK2 cells (Figure 5H). These findings show Smad2 knockdown alleviated cisplatin-induced renal injury, inflammation and programmed cell death *in vitro*.

### Overexpression of Smad2 promoted cisplatin-induced renal injury, inflammation and programmed cell death *in vitro*

**Figure 6:**
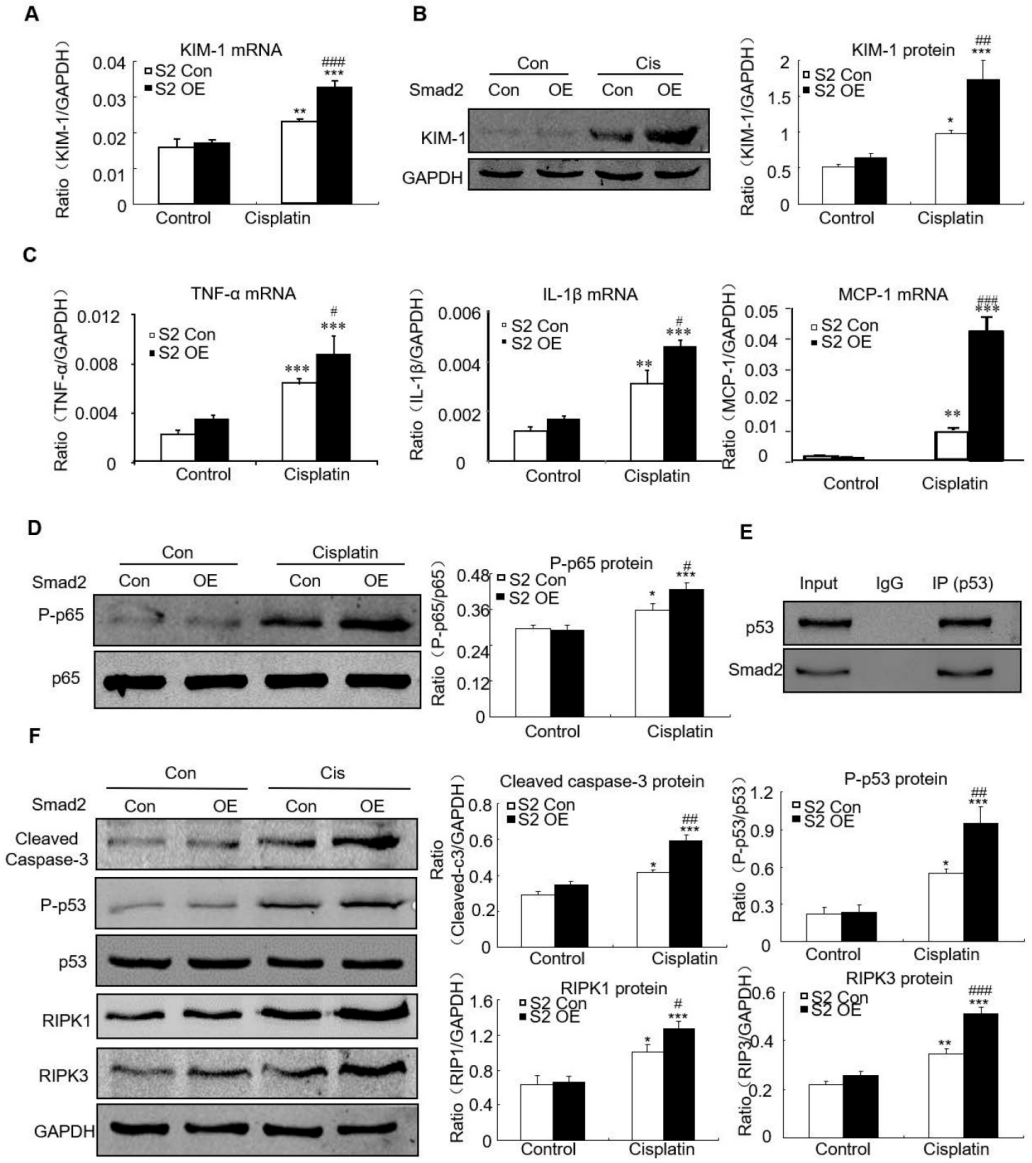
** Overexpression of Smad2 enhanced cisplatin-induced injury, inflammation response and programmed cell death in HK2 cells.** A: Real-time PCR analysis of KIM-1 shows overexpression of Smad2 induced KIM-1 mRNA in cisplatin-treated HK2 cells. B: Western blot and quantitative data of KIM-1 show overexpression of Smad2 increased KIM-1 protein. C: Real-time PCR analysis of inflammation indexes show overexpression of Smad2 enhanced cisplatin-induced inflammation response, including mRNA level of TNF-α, IL-1β and MCP-1. D: Western blot analysis of phospho-p65 in HK2 cells show cisplatin-induced p65 NF-κB phosphorylation was further induced in Smad2 OE cells. E: Co-immunoprecipitation result shows Smad2 physically interacted with p53. F: Western blot analysis of key molecules regulating programmed cell death. Data represent the mean ± SEM for 3-4 independent experiments. *P<0.05, **P<0.01, ***P<0.001 versus control; ^#^P<0.05, ^##^P<0.01, ^###^P<0.001 versus Smad2 vector control+cisplatin. OE: overexpression.

Overexpression of Smad2 enhanced KIM-1 mRNA and protein levels in response to cisplatin compared with Smad2 control vector (Figures 6A and B). Overexpression of Smad2 enhanced cisplatin-induced mRNA levels of inflammatory indexes (Figure 6C). Mechanistically, we found that overexpression of Smad2 promoted cisplatin-induced p65 NF-κB phosphorylation within 1 hour (Figure 6D). The interaction between Smad2 and p53 was validated by using co-immunoprecipitation in HK cells (Figure 6E). Consistently, overexpression of Smad2 increased the key molecules regulating programmed cell death in cisplatin-treated HK2 cells (Figure 6F).

### Lentivirus-mediated Smad2 KD* in vivo* attenuated cisplatin- and I/R-induced kidney injury, inflammation and apoptosis in established AKI mouse models

**Figure 7:**
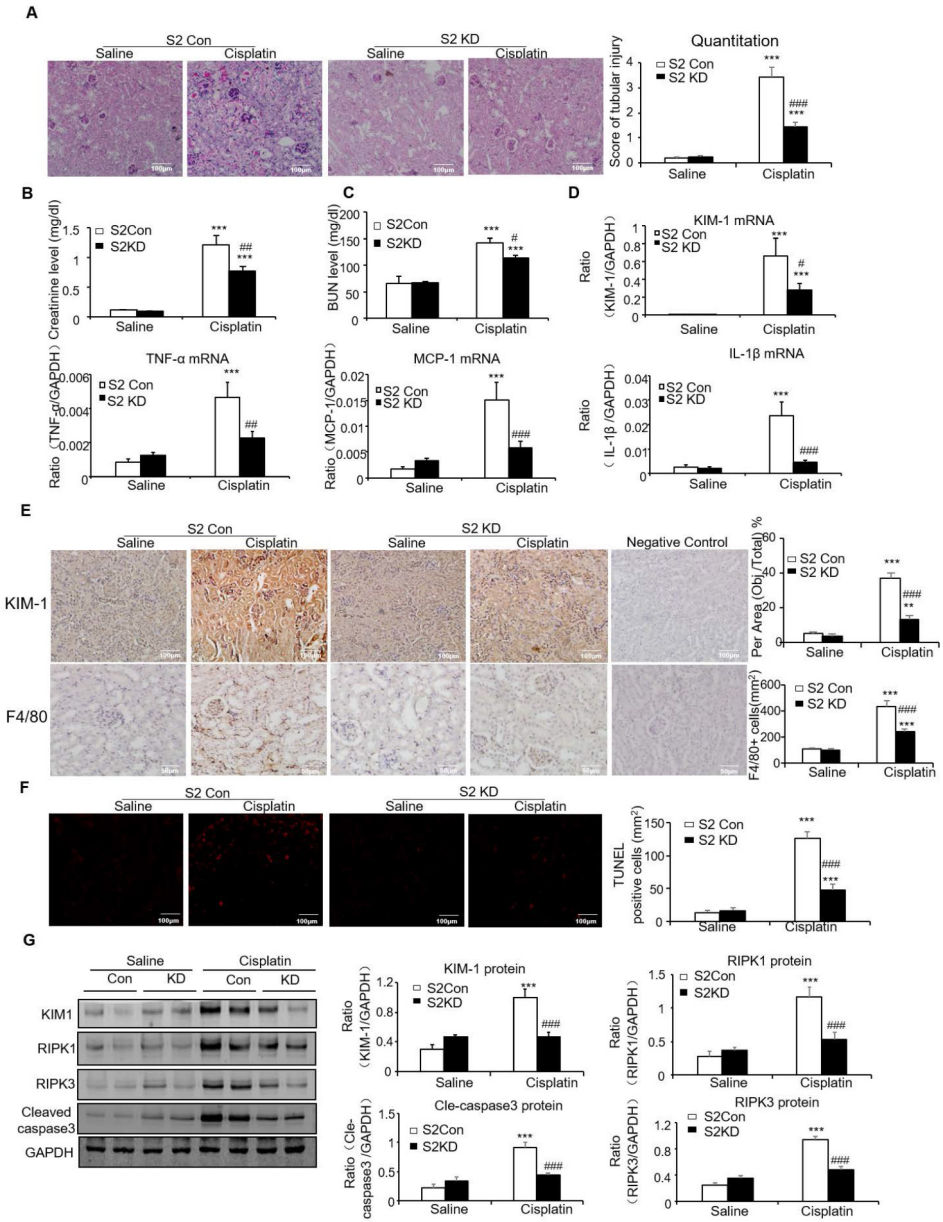
** Knockdown of Smad2 attenuates cisplatin-induced renal injury, inflammation response, and programmed cell death in established nephrotoxic AKI model.** A: PAS staining and quantitative analysis show knockdown of Smad2 reduced renal injury in cisplatin nephropathy. B: Creatinine assay. C: BUN assay. Serum creatinine and BUN show knockdown of Smad2 prevented decline of renal function in cisplatin nephropathy. D: Real-time PCR data show knockdown of Smad2 reduced mRNA of TNF-α, IL-1β, MCP-1 and KIM-1. E: Immunohistochemistry and quantitative data show knockdown of Smad2 reduced KIM-1 protein and F4/80+ macrophages in cisplatin-induced nephropathy. F: TUNEL assay. Knockdown of Smad2 reduced apoptosis in injured kidney. G: Western blot analysis of KIM-1, RIPK1, RIPK3, cleaved caspase-3. Data represent mean ± SEM for 6 mice. *P<0.05, **P<0.01, ***P<0.001 versus control; ^#^P<0.05, ^##^P<0.01, ^###^P<0.001 versus Smad2 vector control+cisplatin. KD: knockdown. Magnification: 100X.

To determine the therapeutic potential of Smad2 in the established AKI mouse model, we silenced Smad2 *in vivo* by tail vein injection of Lentivirus-packaged Smad2 KD plasmid. PAS staining shows Smad2 KD attenuated kidney damage and restored renal function in cisplatin nephropathy (Figures 7A-C).

**Figure 8:**
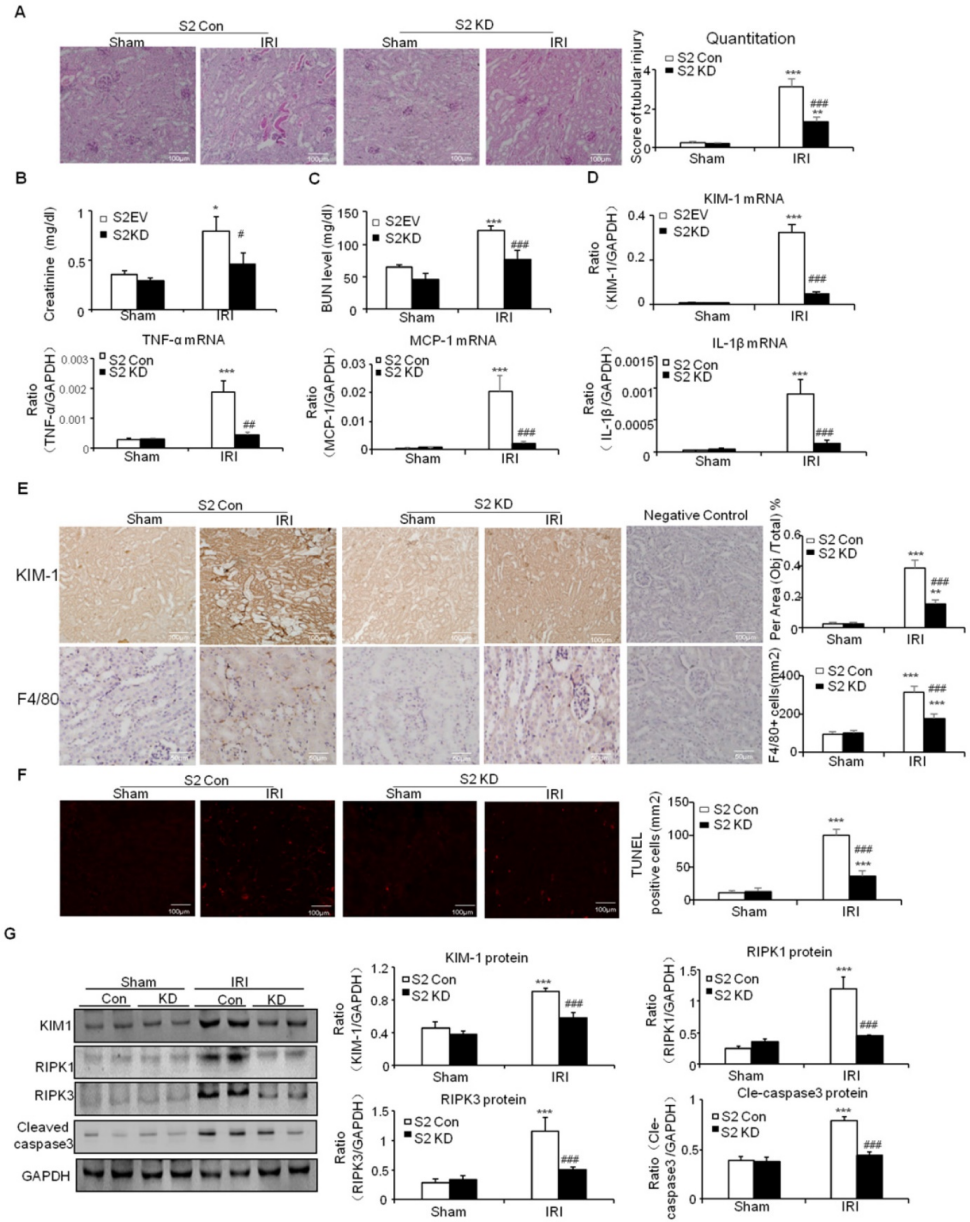
** Knockdown of Smad2 attenuates renal injury, inflammation response, and programmed cell death in established ischemic AKI model.** A: PAS staining and quantitative analysis show knockdown of Smad2 reduced renal injury in I/R-induced AKI model. B: Creatinine assay. C: BUN assay. Serum creatinine and BUN show knockdown of Smad2 prevented decline of renal function. D: Real-time PCR data show knockdown of Smad2 reduced mRNA of TNF-α, IL-1β, MCP-1 and KIM-1. E: Immunohistochemistry and quantitative data show knockdown of Smad2 reduced KIM-1 protein and F4/80+ macrophages infiltration in injured kidney. F: TUNEL assay. G: Western blot analysis of KIM-1, RIPK1, RIPK3, cleaved caspase-3. Data represent mean ± SEM for 6 mice. *P<0.05, **P<0.01, ***P<0.001 versus control; ^#^P<0.05, ^##^P<0.01, ^###^P<0.001 versus Smad2 vector control+IRI. KD: knockdown. Magnification: 100X.

This was further supported by results from real-time PCR and immunostaining of KIM-1 (Figures 7D and E). Moreover, real-time PCR results show that knockdown of Smad2 reduced inflammatory indexes (Figures 7D). This was consistent with the results of Immunohistochemistry of F4/80+ macrophages (Figures 7E). Additionally, TUNEL assay and Western blot results show that knockdown of Smad2 prevented renal apoptosis in established nephrotoxic AKI mouse model (Figures 7F and G). We then evaluated therapeutic effect of Smad2 in the second AKI model induced by I/R injury. Our results consistently show that disruption of Smad2 protected against kidney injury, inflammation and apoptosis in established ischemic AKI mouse model (Figures 8).

### Disruption of Smad2 enhanced cisplatin-induced phosphorylation of Smad3

**Figure 9:**
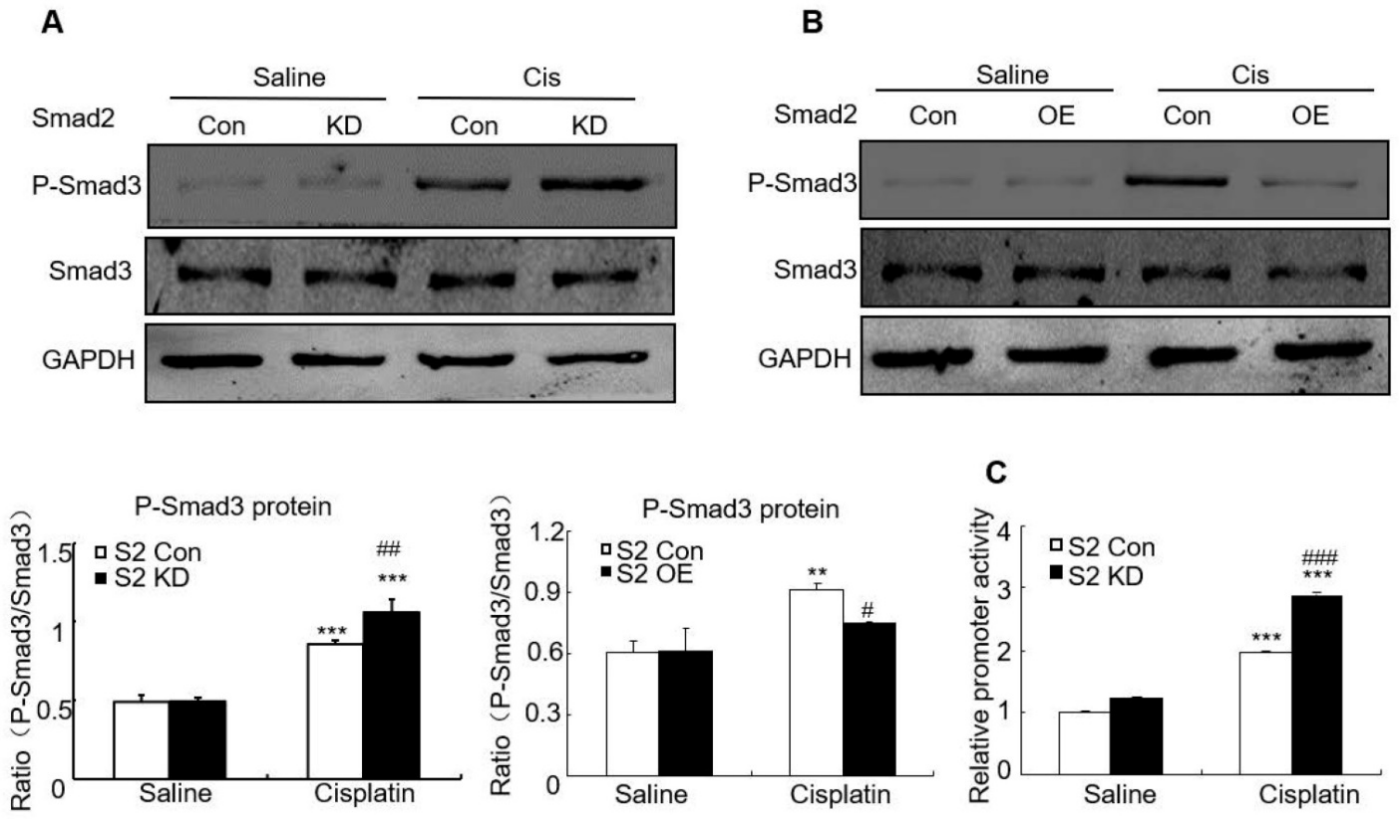
** Effect of Smad2 on cisplatin-induced phosphorylation of Smad3* in vitro*.** A and B: Western blot analysis of phospho-Smad3 in Smad2 KD and OE cells show knockdown of Smad2 increased phosphorylation of Smad3 in cisplatin-treated HK2 cells, which was further confirmed in Smad2 OE HK2 cells. C: Luciferase reporter assay show knockdown of Smad2 promoted cisplatin-induced Smad3 activity compared with control group. Data represent mean ± SEM for 6-8 mice and 3-4 independent experiments *in vitro*. **P<0.01, ***P<0.001 versus normal or control; ^#^P<0.05, ^##^P<0.01, ^###^P<0.001 versus Smad2FF mice or Smad2 vector control+cisplatin. S2FF: Smad2 flox/flox mouse; S2 KspCre: conditional Smad2 knockout mice. KD: knockdown; OE: overexpression.

We then determined whether loss of Smad2 impacted Smad3 signaling in AKI model. Western blot analysis shows that knockdown of Smad2 upregulated Smad3 phosphorylation, but overexpression of Smad2 suppressed Smad3 phosphorylation without altering total Smad3 protein *in vitro* (Figures 9A and B). This is consistent with luciferase reporter assay which demonstrated that knockdown of Smad2 enhanced Smad3 activity in cisplatin-treated HK2 cells (Figure 9C).

## Discussion

TGF-β1/Smad signaling plays a central role in mediating renal fibrosis 3, 24, 25. Although evidence indicates it may function in AKI, the exact role of TGF-β1 and downstream Smads need to be further understood. Here, we generated TGF-βRII conditional knockout mice, Smad2 conditional knockout mice, Smad2 knockdown and overexpression stable HK2 cell lines. We found that disruption of Smad2 in tubular epithelial cells prevented loss of renal function, cell necroptosis, apoptosis and inflammatory response both *in vivo* and *in vitro*. More importantly, lentivirus-mediated Smad2 knockdown protected against renal injury and inflammation while restoring renal function in established nephrotoxic and ischemic AKI models.

In the current study, our result shows that tubular-specific TGF-βRII plays an overall detrimental role in nephrotoxic AKI model. However, some groups found TGF-β1 plays a protective role in acute injury 12, 13, and others have shown accumulation of TGF-β1 induces AKI 14, 15, 26. This discrepancy may be correlated with TGF-β1 level, its cell-type specific role, different types of AKI models or distinct functions of downstream Smad and non-Smad signaling pathways. Moreover, it is of note that downstream Smads may also function in TGF-β-independent manners 3. Given our previous data showing Smad3 promoted fibrosis, while Smad2 and Smad7 resolved renal fibrotic response 25, 27, the complexity and diversity of downstream Smads may have an impact on the overall effect of TGF-β1 in AKI. It's important to note that global knockout of Smad3 limited ischemic AKI by downregulating the production of inflammatory indexes like MCP-1 and IL-6 17. This is consistent with our previous findings that C-reactive protein accelerated AKI by suppressing CDK2/cyclin E in Smad3-dependent mechanisms 28. We also confirmed that disruption of Smad3 protected against cisplatin-induced renal damage and alleviated renal inflammation and apoptosis (unpublished data). These findings suggest Smad3 may be a therapeutic target for AKI. In a recent study, our group showed that Smad7 attenuated ischemic AKI by limiting Smad3-mediated G1 cell cycle arrest of TECs 18. Given the role of Smad2 in AKI is not well described, we conditionally knocked out Smad2 from kidney TECs and induced AKI by intraperitoneal injection of cisplatin. Results show creatinine and BUN levels decreased. And, PAS staining show renal damage was alleviated.

We also found that knockout of Smad2 from TECs attenuated cisplatin-induced programmed cell death in mice. Proximal TECs are the primary target of AKI, and programmed cell death of TECs is a common feature for different types of AKI caused by ischemia reperfusion injury and nephrotoxic insult 29, 30. As an important regulator of kidney injury and repair, TGF-β1 is highly involved in apoptosis and proliferation of TECs. Previous studies showed that inhibition of TGF-β1 reduced TECs apoptosis in chronic renal fibrosis 31, 32. Further, TGF-β1 initiated TECs apoptosis via P38/ERK signaling instead of Smad signaling 33, 34. In contrast, other studies showed that Smad2/3 signaling triggers apoptosis by regulating cell cycle repressor elements or Bcl-2 family 35, 36. This indicates TGF-β1 induces apoptosis in both Smad-dependent and Smad-independent mechanisms depending on different disease conditions. Of note, role of Smad2 in nephrotoxic agent-induced apoptosis in AKI is still unknown. In the current study, our results show that knockdown of Smad2 decreased the percentage of apoptotic TECs, cleaved caspase-3 level, p53 phosphorylation and activity. Moreover, overexpression of Smad2 promoted apoptosis in HK2 cells possibly by binding to p53 with the help of co-activators, which was further confirmed *in vivo* in Smad2 conditional knockout mice. This was consistent with previous study demonstrating that Smad/p53 functional interactions were associated with renal fibrosis 37, 38. We noticed that disruption of Smad2 also alleviated cell necroptosis in cisplatin nephropathy. Necroptosis is the best-known pattern of regulated necrosis, emerging evidence showed RIPK1, RIPK3 and MLKL, central regulators in necroptotic pathway, play significant roles in mediating AKI 39, 40. Our team recently found that by binding to the ATP-binding pocket of RIPK1, wogonin prevents cisplatin-induced necroptosis and renal injury 22. We also identify hsa-miR-500a-3P directly target the 3'UTR of MLKL, thereby alleviates kidney injury via limiting necroptosis 41. Compared with apoptosis, necroptosis incurs more severe consequence because cells suffered necroptosis release endogenous pro-inflammatory molecules like damage-associated molecular patterns (DAMPs) and promote renal inflammation 42, 43. In the current study, our results showed that loss of Smad2 prevented, but overexpression of Smad2 promoted, cisplatin-activated necroptotic signaling, this result indicates Smad2 may play an important role in mediating cell necroptosis and consequent necroinflammation.

Additionally, we show disruption of Smad2 decreased cisplatin-induced inflammatory response both* in vivo* and *in vitro*. Overproduction of cytokines and recruitment of macrophages are major features of AKI 44, but the role of TGF-β1/Smads in renal inflammation remains disputed. Smad3 confers a chemotactic effect by inducing the transcriptional activity of MCP-1 and macrophage infiltration. In contrast, TGF-β1 and Smad3 exert anti-inflammatory effects on TNF-α-induced renal inflammation 45, 46. We previously found that conditional knockout of TGFβRII or Smad4 increased renal inflammation by abolishing the anti-inflammatory effect of Smad3 20, 45. In the current study, conditional knockout of Smad2 downregulated cisplatin-induced inflammatory cytokine production and NF-κB activity, this may be led by overactivation of Smad3. Of note, the effect of TGF-β1/Smads on inflammation may be highly dependent on TGF-β1 concentration, disease types and cell types. These factors need to be further examined.

We also investigated the effect of Smad2 deficiency on Smad3 signaling in cisplatin-induced AKI. Although Smad2 and Smad3 have more than 90% homology in amino acid sequence and interact physically, their functions differ in renal fibrosis 19. We found that disruption of Smad2 induced smad3 phosphorylation and activity in cisplatin-treated TECs. This is consistent with our previous study that showed loss of Smad2 promoted Smad3 signaling in fibrotic kidney 19. However, unlike the diverse role of Smad2 and Smad3 in renal fibrosis, blocking Smad2 or Smad3 is capable to attenuate AKI. This indicates Smad2 cooperates with Smad3 in mediating programmed cell death and kidney injury.

Finally, we evaluated the therapeutic potential of Smad2 in established AKI mouse models. It is noteworthy that Smad2 knockdown protected against kidney injury, programmed cell death and renal inflammation in both nephrotoxic and ischemic AKI mouse models.

**Figure 10:**
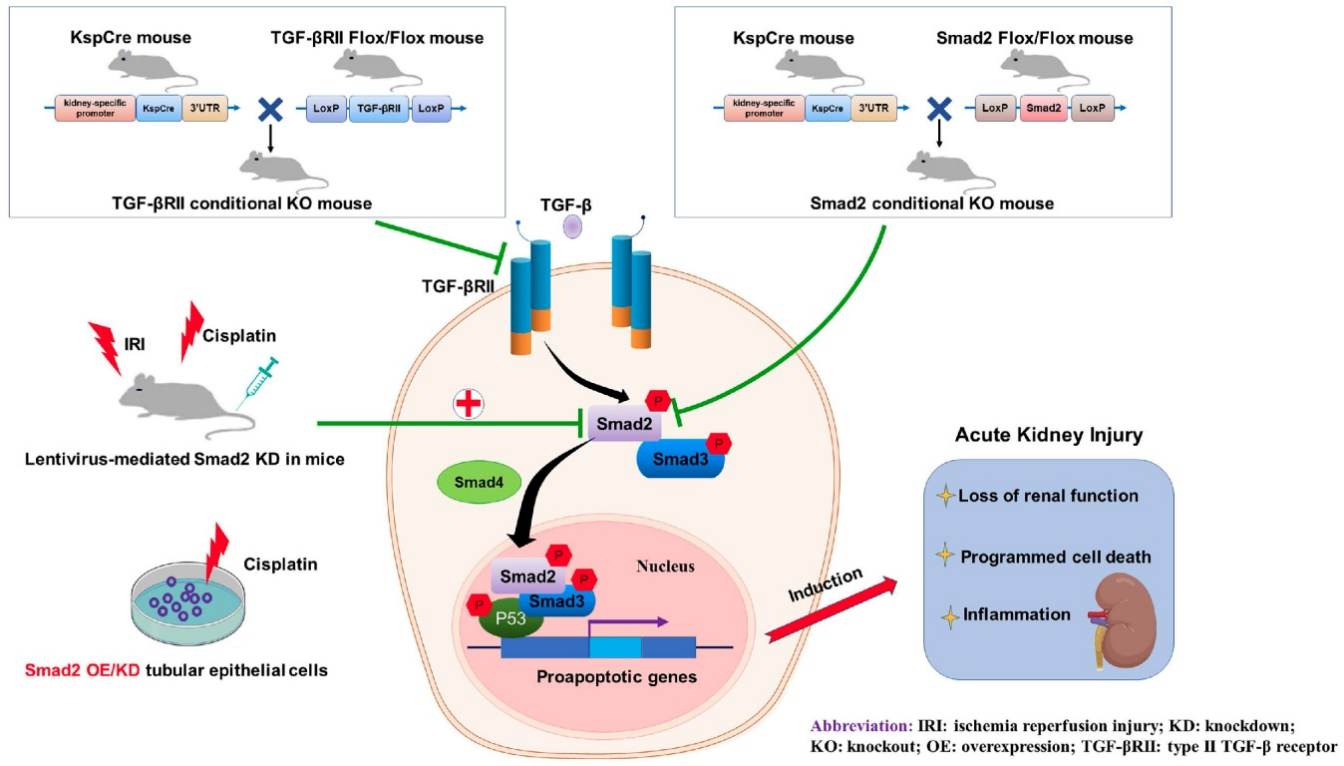
** Role of TGF-βRII and Smad2 in acute kidney injury.** Conditional knockout of TGF-βRII or Smad2 from kidney protects against AKI by alleviating cell necroptosis, apoptosis and inflammation, and Smad2 may serve as a therapeutic target for nephrotoxic and ischemic AKI.

In conclusion, we found that TGF-β promoted renal damage in AKI model, and disruption of Smad2 attenuated cisplatin and I/R-induced kidney injury via reducing programmed cell death of TECs and p65 NF-κB-driven renal inflammation (Figure 10). Therefore, Smad2 may serve as a potential therapeutic target in AKI treatment.

## Abbreviations

AKI: acute kidney injury
HK2: Human renal tubular epithelial cells
IL-1β: interleukin-1 beta
I/R: ischemia/reperfusion
KIM-1: kidney injury molecule-1
LAP: latency-associated peptide
LTBP: Latent TGF-β-binding protein
MCP-1: monocyte chemotactic protein-1
MLKL: mixed lineage kinase domain like pseudokinase
RIPK: Receptor-interacting serine/threonine-protein kinase
S2FF: Smad2 flox/flox mouse
S2 KspCre: conditional Smad2 knockout mice
S2KD: Smad2 knockdown
S2OE: Smad2 overexpression
TECs: renal tubular epithelial cells
TGF-β: Transforming growth factor-β
TNF-ɑ: Tumor Necrosis Factor
TGF-βRII FF: TGF-β type II receptor flox/flox mouse
TGF-βRII KspCre: conditional TGF-β type II receptor knockout mice
